# Cornual pregnancy as a complicaton of the use of a levonorgestrel intrauterine device: a case report

**DOI:** 10.4076/1752-1947-3-8387

**Published:** 2009-07-22

**Authors:** JJ Beltman, CJM de Groot

**Affiliations:** 1 Medisch Centrum Haaglanden, Department of Obstetrics and Gynaecology, Lijnbaan 32, 2501 CK, The Hague, The Netherlands

## Abstract

**Introduction:**

Complications of copper load intrauterine devices, including ectopic pregnancies are well reported. Rates of ectopic pregnancy are 0.6 to 1.1% per year. However, the levonorgestrel intrauterine device has been described as more protective against ectopic pregnancies due to the addition of the hormone levonorgestrel. The hormone released from the intrauterine device causes some systemic effects, but local effects such as glandular atrophy and stromal decidualization, in addition to foreign body reaction, are dominant. Few case reports have described ampullary ectopic pregnancies. However, we report, for the first time, a major complication of levonorgestrel intrauterine device: a cornual pregnancy.

**Case presentation:**

A 36-year-old Caucasian nulliparous woman presented with complaints of progressive nausea, abdominal pain and irregular vaginal bleeding for 2 months. For 3 years, she had been using a levonorgestrel intrauterine device. A two-dimensional transvaginal sonogram noted a sac situated external to the endometrial cavity in the right cornua of the uterus with an empty uterus. She was successfully treated with chemotherapy.

**Conclusion:**

Many complications have been described, including ectopic pregnancies, using copper intrauterine devices. The levonorgestrel-releasing intrauterine system is a particularly good choice for adolescents because of associated non-contraceptive benefits such as decreased menstrual bleeding, dysmenorrhea and pain associated with endometriosis [1]. Yet a cornual pregnancy following the use of a levonorgestrel intrauterine device is a complication which, to our knowledge, has not been described before. Physicians prescribing this type of intrauterine device should be aware of this rare event.

## Introduction

Many studies have described ectopic pregnancies as a complication of copper intrauterine devices (IUDs) while other studies suggest the protective role that levonorgestrel IUDs have on the incidence of ectopic pregnancies. In our patient, a cornual pregnancy occurred using a levonorgestrel IUD.

## Case presentation

A 36-year-old Caucasian nulliparous healthy woman was referred to our outpatient clinic with a positive pregnancy test and complaints of progressive nausea, abdominal pain and irregular vaginal bleeding for 2 months. For 3 years, she had been using a levonorgestrel-releasing intrauterine device as a contraceptive, which was removed by the general practitioner the same day. She had no history of prior ectopic pregnancy, pelvic inflammatory diseases or previous tubal-uterine surgery, in vitro fertilization or other assisted reproduction procedures. She did not smoke.

**Figure 1:**
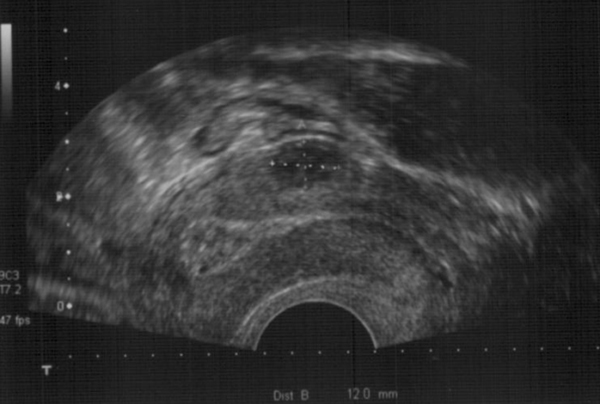
**(A) Transvaginal ultrasound of the cornual pregnancy. (B) Detailed clarification of the ultrasound in (A)**.

**Figure 2:**
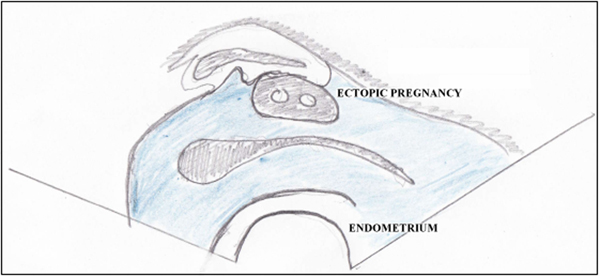
**(A) Transvaginal ultrasound of the cornual pregnancy. (B) Detailed clarification of the ultrasound in (A)**.

On examination, her blood pressure was 108/64 mmHg and she was afebrile. There was slight tenderness in her right lower abdominal quadrant. No guarding or rebound tenderness was noted, and no abdominal mass was palpable. Laboratory findings were: quantitative human chorionic gonadotropin (hCG) level of 69,030 mIU/mL, hemoglobin 7.9 g/dL (12.1-15.3 g/dL), white cell blood count 6.5 × 10^9^/L and normal liver chemistry test. A two-dimensional transvaginal sonogram revealed a sac situated external to the endometrial cavity in the right cornua of the uterus (>1 cm from the most lateral edge of the uterine cavity) containing an embryo measuring 5 mm with positive heart rate consistent with a 6-week pregnancy. The sac had a thin surrounding myometrial layer. Neither free fluid nor adnexal mass were noted (Figure 1). Chlamydia tests on admission were negative. After informed consent, chemotherapy was preferred by the patient to either dilatation and curettage or laparotomy to preserve fertility. She was treated as having an ectopic, cornual pregnancy with chemotherapy. Four doses of intramuscular methotrexate (1 mg/kg) were administered followed by leucovorin (0.1 mg/kg) on alternate days to enhance destruction of trophoblastic tissue. In the following 2 weeks, the quantitative hCG level declined to 64% of its original value (Figure 2).

**Figure 3:**
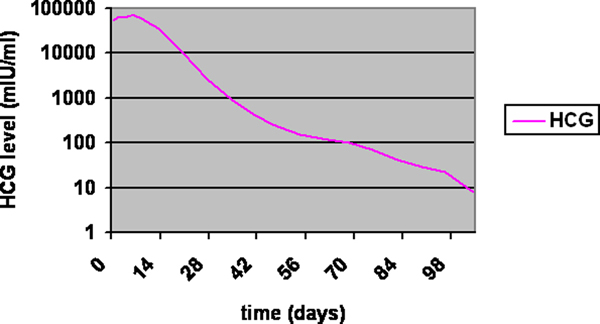
Human chorionic gonadotropin levels during treatment in days.

The patient was discharged from hospital after signs of resolution of the ectopic pregnancy and was seen for follow-up until hCG levels were <5 mIU/mL and follow-up ultrasound findings did not reveal any abnormalities: normal uterine cavity, no adnexal mass, no abdominal fluid. Hysteroscopic evaluation showed the same: no abnormalities, both uterine openings open to fallopian tubes. There were no side effects to the methotrexate treatment.

## Discussion

Ectopic pregnancies account for 1.5% of all reported pregnancies in western countries [2]-[4]. The terms 'cornual pregnancy' and 'interstitial pregnancy', both types of ectopic pregnancy, are used in the medical literature interchangeably. By definition, a cornual pregnancy refers to the implantation and development of a pregnancy in the lateral upper portion of the uterus, whereas an interstitial pregnancy implants within the myometrium of the proximal and intramural portion of the fallopian tube [5].

Most of the risk factors of ectopic pregnancies are known. The most common risk factors are: history of pelvic inflammatory disease, smoking, induction of ovulation, history of ectopic pregnancy, previous pelvic surgery and the use of IUDs [5]. Whether salpingitis associated with IUD use is a cause of ectopic pregnancy or whether the IUD prevents intrauterine pregnancies but not ectopic pregnancies remains unknown.

The prevalence of ectopic pregnancies and IUD use differs between studies. In France and Norway, women with IUDs accounted for 25-30% of all ectopic pregnancies [6,7], whereas in a British study, IUDs accounted for only 14% [8]. These differences may result from differences in the rate of IUD use, differences in the kind of IUD and differences in adequate registration.

Almost all of the literature concerning ectopic pregnancies and IUD use relate to copper IUDs rather than levonorgestrel IUDs. Data on levonorgestrel IUD use complicated by ectopic pregnancies are limited. Only a few studies have investigated the relationship between ectopic pregnancies and levonorgestrel IUD use: in a 5-year follow-up, international comparative study between levonorgestrel-20 and TCu-380(Ag), none of the six pregnancies in 1124 women with the levonorgestrel-IUD-20 were ectopic. In women using the Copper T 380 (*n* = 1121), two ectopic pregnancies occurred, with a 5-year ectopic pregnancy rate of 0.6 per 1000 woman-years [9].

In another study that compared the levonorgestrel-IUD-20 with the Nova-T device, after one year of use, the only pregnancy occurring in levonorgestrel-IUD-20 users (*n* = 1821) was intrauterine, and one of the eight pregnancies occurring in Nova-T users (*n* = 937) was ectopic. The 3-year ectopic pregnancy rates per 1000 women were 0.3 among the former and 2.5 among the latter (*p* = 0.02) [10].

The above studies suggest that the levonorgestrel IUDs may have a protective effect against the incidence of ectopic pregnancy. But there are more advantages: in addition to the long-acting, safe and reversible method of contraception, it also offers a protective effect on pelvic inflammatory disease and has a high level of acceptability with the majority of women requesting effective contraception [11]. However, this case report shows an ectopic pregnancy with levonorgestrel IUD use. In addition, to our knowledge, this is the first case report describing an ectopic, cornual pregnancy with levonorgestrel IUD use.

Although non-surgical treatment of cornual pregnancies faces higher failure rates than treatment of ampullary pregnancies [12], we adopted this non-surgical management since there was no acute emergency (of a ruptured ectopic pregnancy). In addition, a surgical approach would have had an increased risk of severe hemorrhage with salpingectomy and opening the uterus with possible negative consequences for future pregnancies. Several reports have recommended treating an interstitial pregnancy conservatively. Jermy et al. [13] suggested that systemic methotrexate is a safe and highly effective treatment for interstitial pregnancy. Surgery can be avoided in the majority of women with this condition. Early recognition of the cornual pregnancy with transvaginal ultrasound is essential. Surgery means cornual resection or hysterectomy thereby compromising reproductive function [13]. Curettage was not considered because of the ectopic aspect from ultrasound.

## Conclusion

A nulliparous woman presented with a cornual pregnancy, consistent with 6 weeks of gestation and with cardiac activity. She had been using a levonorgestrel IUD. She was treated successfully with conservative management. One of the reasons not to treat the patient surgically was to avoid severe hemorrhage and to preserve reproductive function.

## Consent

Written informed consent was obtained from the patient for publication of this case report and any accompanying images. A copy of the written consent is available for review by the Editor-in-Chief of this journal.

## Competing interests

The authors declare that they have no competing interests.

## Authors' contributions

JJB collected and analyzed the patient data and wrote the manuscript. CdG interpreted the data regarding cornual pregnancy and was a major contributor in writing the manuscript. Both authors approved the final manuscript.
